# A cross-sectional observational study of birefringent particulates in bronchoalveolar lavage cytology in horses with equine asthma from the West v East coasts of the USA

**DOI:** 10.1371/journal.pone.0297181

**Published:** 2024-04-04

**Authors:** Melissa R. Mazan, Edward F. Deveney

**Affiliations:** 1 Department of Clinical Sciences, Tufts University Cummings School of Veterinary Medicine, Grafton, MA, United States of America; 2 Department of Physics, Photonics and Optical Engineering, Bridgewater State University, Bridgewater, MA, United States of America; Novartis Institutes for BioMedical Research, UNITED STATES

## Abstract

Equine asthma (EA) is an important cause of wastage in the USA horse industry. Exposure to organic particulates, from stable dust, airborne pollen, and fungal loads, is posited to be the main cause. Dust arising from the earth’s crust has been largely ignored as a contributor to EA in the veterinary literature. The objectives of this study were to investigate the occurrence of birefringent particulates in the bronchoalveolar lavage fluid (BALF) of horses with a clinical complaint of EA residing in the arid West of the USA v. the East, in an effort to determine the contribution of geolocation to geogenic dust exposure. We analyzed BALF cytology and historical data sent to our referral clinical laboratory from 148 horses from the West Coast and 233 horses from the East Coast of the USA over a 6-year period, using light microscopy to determine cell proportions and other visible elements as well as a polarizing lens to detect birefringent material. Univariate analysis showed that horses from the West coast were significantly more likely to have birefringent particulates in the BALF than horses from the East coast (40.5% v. 8.6%, *p* < 0.001); while horses from the East had higher BALF neutrophil proportions. Horses from the West also had lower proportions of neutrophils in the BALF than those from the East (27.1 v. 10.9, *p* < .001). Using historical and BAL data in a forward stepwise binary logistic regression model with presence of birefringent particulates found within alveolar macrophages as the outcome, geographical location in the West retained significance as a predictor (OR 8.0, CI [4.3–14.8], p< .001). While the birefringent particulates cannot be identified on the basis of polarizing microscopy alone, this study provides evidence that horses from the West are exposed to inorganic particulates that may contribute to signs of equine asthma.

## Introduction

Equine asthma (EA), a non-septic inflammation of the lower airways that causes a wide spectrum of clinical signs varying from subtle poor performance to chronic cough and repeatable episodes of respiratory embarrassment, results in significant wastage in the horse industry throughout the world. The diagnosis of equine asthma is established by a history of chronic cough unaccompanied by fever, and poor performance or exercise intolerance that frequently is connected to exposure to organic dust, prior viral and bacterial infection, or genetic factors [1]. Lung function testing, which can only be done in specialized centers, reveals airway hyperreactivity and, in severe cases, elevated respiratory resistance. The relationship between lung function, athletic performance, and inflammatory cell proportions in bronchoalveolar lavage fluid (BALF), however, have been established in horses with mild to severe asthma. Unlike humans, in which bronchoalveolar lavage (BAL) requires a bronchoscopy suite, is considered invasive, and is reserved for the diagnosis of interstitial disease, the most common diagnostic method for equine asthma is retrieval of BAL fluid for cytologic examination [1]. While organic dust is highly linked to the initiation and exacerbation of equine asthma, the role of inorganic particulates found in geogenic dust has been largely ignored, despite BAL fluid being highly accessible as a means of assessing exposure to inorganic dust in horses.

As global warming creates arid and semi-arid climates over increasingly large areas of the earth, there is increased interest in understanding the role of inorganic dusts derived from the earth’s crust in causing or exacerbating respiratory disease in humans [2]. While the extremes of outdoor dust exposures, labeled dust events or desert dust storms, are well-documented causes of increased hospital admissions and even mortality for asthma, COPD, and cardiovascular disease in people [3] and occupational exposure to silicates, such as seen in talc workers [4] is known to cause pneumoconiosis and silicosis in humans, there is also a considerable but less extreme quotidian outdoor exposure to inorganic particulates, known as ‘aolian’, geogenic, or desert dust of crustal origin, in arid parts of the world, including the West coast of the USA [5]. Higher exposure to geogenic (earth-derived) dust in arid regions is a strong risk factor for both allergic and infectious respiratory illness in indigenous children in Australia [6, 7], and farmers exposed to high levels of geogenic dust in arid regions also have an increased risk of both infectious and non-infectious disease [1]. Moreover, there has been recognition and documentation that geogenic dust causes a disproportionate burden of respiratory disease in people in disadvantaged areas of the world [6]. There is potential for the horse, an outdoor-dwelling animal with high respiratory flows, to help bridge the gap in our knowledge of the role of geogenic dust in respiratory disease, thus making this an important area of study for both humans and animals.

Silica and silicates comprise approximately 90% of the earth’s crust, making them a good marker of exposure to geogenic dust. It is convenient that silica and silicates are highly birefringent under polarizing microscopy, which renders them also available for detection in clinical samples [8]. In order to understand the usefulness of polarizing microscopy, a brief review is useful. When materials with an Isotropic index of refraction are driven by polarized light they reradiate in the same direction of polarization at which they were initially driven. In contrast, anisotropic materials, such as birefringent crystals, can reradiate polarized light in directions different to those at which they were driven. This principle can be employed using light microscopy: here, source light is linearly polarized in the plane of the sample which can be taken as horizontal. Isotropic material reradiates in that same horizontal polarized direction, whereas an anisotropic material, such as birefringent crystalline material, will reradiate the driving horizontally polarized light into directions other than horizontal. A second linear polarizer is used to filter the reradiated light from the sample. When the orientation of that second filter is parallel to the orientation of the first (preparation) filter defined as horizontal, then for isotropic material, light is visible. As the second filter is rotated away from the horizontal, light is extinguished. If, on the other hand, there are anisotropic, possibly crystalline particulates in the sample, they will reradiate the incoming polarized light into many different directions that can be observed when the probe filter is rotated away from horizontal. Indication of anisotropic and possible birefringent crystalline material is identified by illumination within the otherwise dark viewing field at probe linear polarization angles other than that which matches the preparation driving excitation angle (horizontal). Consequently, while the precise identification of mineral material found in respiratory secretions requires more sophisticated and complex analysis, the presence of anisotropic minerals that are commonly found in dust from the earth’s crust can also be seen using simple light microscopy by noting their characteristic birefringence through use of polarized light.

Polarizing lens microscopy has been used in humans to document birefringent particulates for the investigation of both occupational and environmentally mediated mineral dust causes of respiratory disease, but this modality is seldom employed in analysis of bronchoalveolar lavage cytology in animals. Our cross-sectional observational study cannot determine the cause of equine asthma in geographically separate populations from the arid West of the USA v. the East; instead, it aims to determine whether geographic differences in exposures can be detected, and for the first time, to determine if horses in an arid environment have evidence of a different exposure and response to airborne exposures in comparison to horses from a non-arid environment. The focus of this study is to compare bronchoalveolar lavage cytologies, including cell proportions, presence of birefringent particulates, and presence of fungal elements or pollen, as well as clinical presentations of horses living on the West Coast v. the East Coast of the United State and presenting with a suspicion of respiratory disease, in order to determine if there are differences in BAL cytology and disease presentation between these two populations.

## Methods and materials

### Study design

Bronchoalveolar lavage reports from samples sent to the Equine Respiratory Health Laboratory at Tufts Cummings School of Veterinary Medicine over a 6-year period (2018 through 2023) for which history, clinical examination, and bronchoalveolar lavage cytology, including polarizing microscopy, were available, were used for a retrospective cross-sectional study of BAL cytology using STROBE-guidelines [9] (n = 643). All samples were acquired from privately owned animals in the USA in order to detect suspected disease and therefore improve health, and all veterinarians had a recognized veterinary-client-patient relationship (VCPR), therefore no IACUC approval was required. All records were examined to determine the geographic location of the referring practice, as well as the horse’s clinical complaint, current use, sex and breed, as well as documentation of proportions of cells and number of fungal elements per high power field, and particulates seen with polarizing microscopy. Cases were categorized as well by season, with June, July and August considered summer, September, October, and November considered autumn, December, January and February considered winter, and March, April and May considered spring. Cases were further designated by the location of the referring practice as being from the West or the East of the United States according to the USA Department of Labor, and all case records from practices located in Western states, represented by California, Arizona, Utah, South Dakota, Montana, Colorado, Oklahoma, Oregon, North Dakota, Nevada, Idaho, New Mexico, Texas, Washington State, and Wyoming (n = 157) and Eastern states represented by Connecticut, Maine, Massachusetts, Maryland, New Jersey, New York, Pennsylvania, Rhode Island, Vermont, Virginia, West Virginia, and Florida (n = 234) were selected for a total of 391 cases.

**Fig 1:**
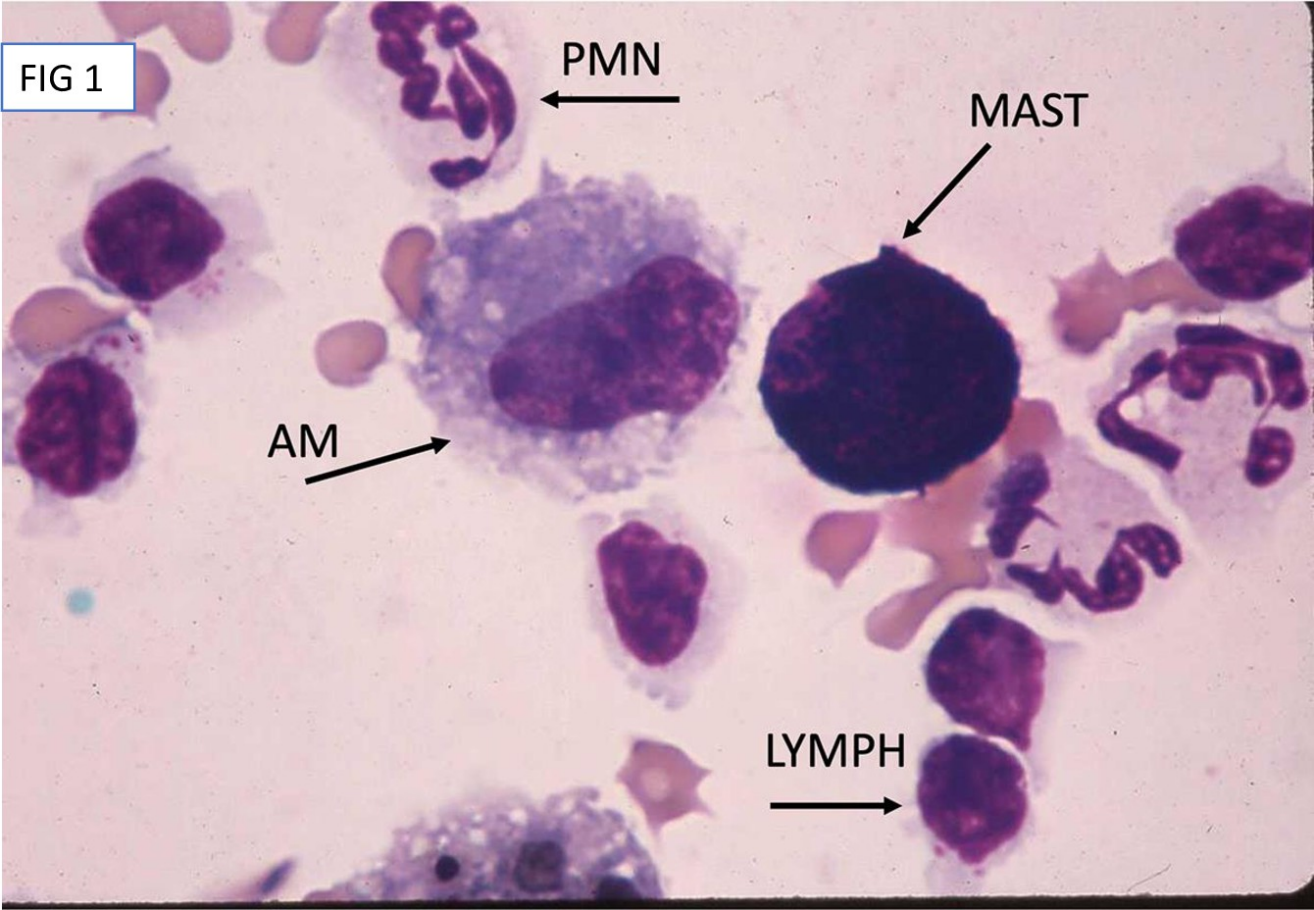
High magnification image of representative cells in BALF. Sample was prepared via sedimentation, stained with a modified Wright-Giemsa (Diff Quick) and viewed using light microscopy at 1000x under oil. Representatives of cell types are labeled as AM (alveolar macrophage), LYMPH (lymphocyte), PMN (polymorphonuclear neutrophil), and MAST (mast or metachromatic cell).

**Fig 2:**
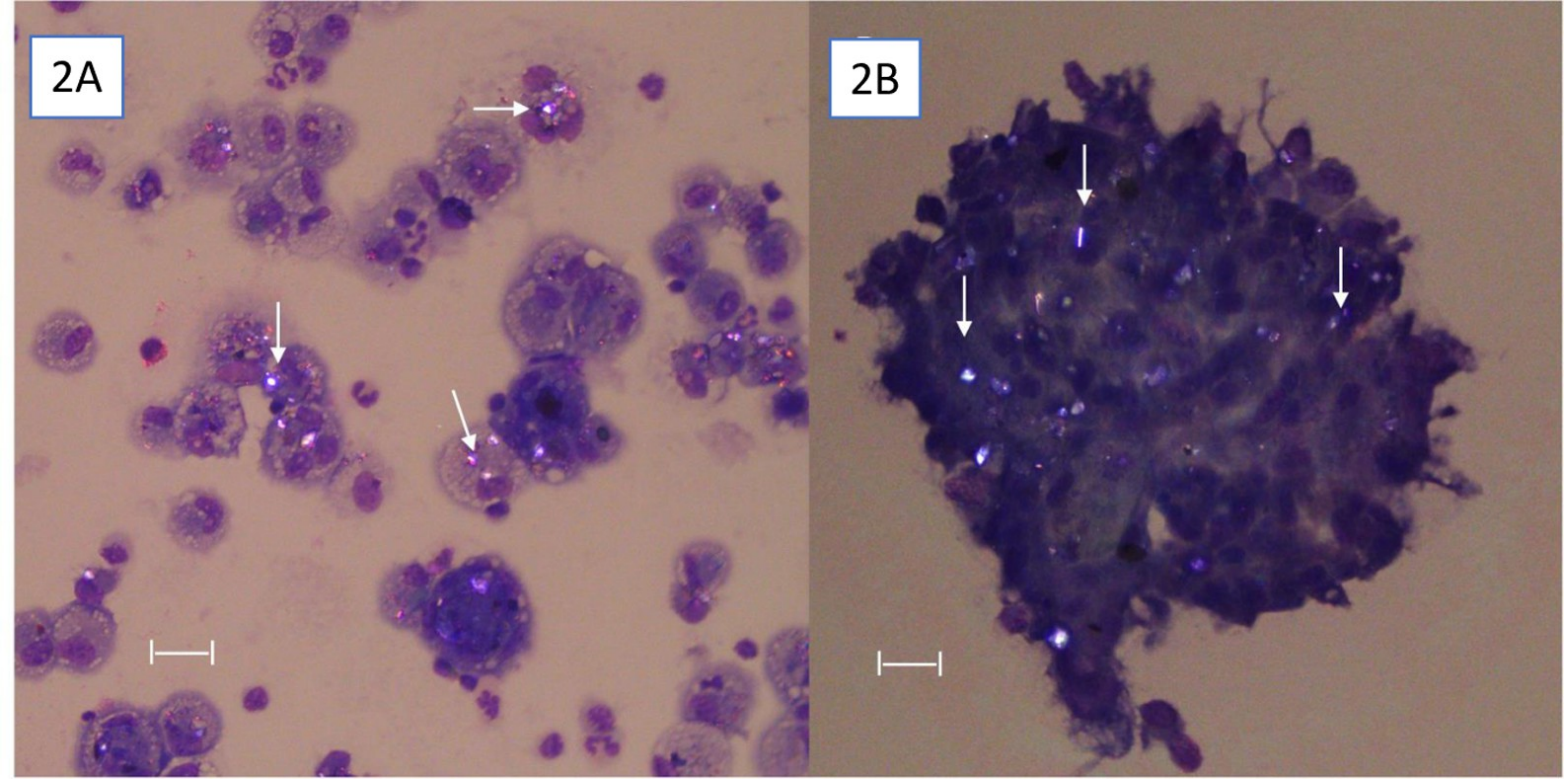
Birefringent particulates in bronchoalveolar lavage fluid cytology. Samples were prepared via sedimentation, stained with a modified Wright-Giemsa (Diff Quick) and viewed using polarizing microscopy at 400x. White arrows indicate birefringent particulates. (A) Multiple alveolar macrophages contain birefringent particulates within the confines of and at the same depth as the cytoplasm. (B) Large, bizarre multinucleated giant cell containing many birefringent particulates. Bar = 20 um. Original magnification 400x under oil.

BAL cytologies were received as fluid in EDTA preservative tubes, sent on ice by overnight mail (95% of submissions), or as slides made by sediment smear at the referral site (<5% of submissions). Fluid was centrifuged at 500g for 12 minutes, the supernatant was poured off, and the remaining pellet was resuspended to allow concentrated cells to remain. A transfer pipette was used to place an approximately 50 ul drop on the frosted end of a slide, and a ‘puller’ slide was used to bring the suspension ½ to 2/3rds of the way to the end of the slide, leaving a consistent line of concentrated but non-overlapping cells, and the slide was rapidly air-dried. Slides were stained with modified Wright-Giemsa and toluidine blue, and for all BAL cytology analyses, a 500-cell count was used to determine cell proportions. Alveolar macrophages were identified as larger (20–50 um in diameter) cells with oval nuclei and either finely stippled, foamy, or vacuolated cytoplasm. Both larger and smaller lymphocytes are present in equine BAL cytology, ranging from 8um to 20 um in diameter, with a dark, round nucleus and homogenous cytoplasm. Neutrophils were identified by their polymorphonuclear appearance, eosinophils by the strongly eosin-staining, large granules, and mast cells were identified by their metachromatic granules seen on toluidine blue staining (Fig 1). The presence of particulates including fungal elements at 400x under oil immersion was also noted and quantified. Proportions of alveolar macrophages, lymphocytes, neutrophils, and eosinophils were determined on a first count of Diff-Quik stained cytology, and then epithelial cell percentage was counted separately. Proportion of mast cells was determined separately on toluidine blue-stained slides. The most recent consensus statement of the International Equine Asthma Group was used to determine inflammation of the lower airways, with normal macrophage proportions considered to be 40–60% of the total cell population, lymphocytes 40–60% of the total cell population, neutrophils < or equal to 5% of the total cell population, mast cells < or equal to 2% of the total cell population, and eosinophils < 1% of the total cell population [1]. Fungal elements were seen most commonly as single-celled spores or rarely as fragmented hyphae, and were noted as 1, 2–4, or > 4 per high power field. Particulates seen within the confines of cells and extracellularly were also noted. The entirety of the slide was then assessed using a polarizing lens at 200x for an initial overview of the presence of birefringent particulates, and then entire slide was assessed at 400x to determine number of alveolar macrophages containing birefringent particulates. All birefringent material was noted, but only particulates that were within the visible confines of the cytoplasm of alveolar macrophages and were in the same plane were counted as intracytoplasmic to avoid potential artifact. Birefringence was classified as none seen, with a score of 0; extracellular and rare (less than 5% of alveolar macrophages) intracytoplasmic, with a score of 1; occasional (5–10% of alveolar macrophages) intracytoplasmic and extracellular, with a score of 2; or many (> 10% of alveolar macrophages) intracytoplasmic and extracellular particulates, with a score of 3 (Fig 2A).

### Statistical analysis

All data are presented descriptively with continuous variables reported as mean ± standard deviation or median and range in the case of non-normal distribution, and categorical data are presented as frequency and percentage. Univariate statistical analyses were based on the normality of data distribution (Kolmogorov–Smirnov test) and employed independent samples *t*-test, Mann–Whitney *U*-test, and Pearson chi-square analyses to compare outcome groups and to assess differences among populations (accepted significance level, *P <* 0.05, or adjusted residual absolute value > 1.96). Additionally, univariate and stepwise multinomial logistic regression were performed for both continuous and categorical variables, if documented for at least 80% of horses in the submission information. These were used to derive odds ratios (ORs) for analysis of outcome, using standard criteria of 0.05 for entry and 0.2 for removal. All data were analyzed using commercially available statistical software (IBM SPSS Statistics 28.0, Chicago, Ill 60606).

## Results and discussion

Horses from the West and East Coasts of the United States differed in their clinical presentation on univariate analysis, (Table 1) with horses from the East coast significantly older, and with a significantly greater proportion of geldings than mares. More horses from the West than the East coast were used for barrel racing and other Western sports as well as racing, while more from the East were used for dressage, driving, pleasure, and show. There was no significant difference in the proportion of horses used for hunter/jumper or endurance. Quarter Horses and Thoroughbreds were over-represented in the West, whereas Warmbloods, Standardbreds, Drafts, Miniature Horses, and Morgans were. overrepresented in the East. Cough was the most commonly noted clinical complaint in both populations, with horses from the East more likely to cough (75.6% v. 59.2%), followed by exercise intolerance, and, least frequently, nasal discharge. There was no significant difference in the time of year at which the BAL was performed.

**Table 1:** Patient characteristics (mean and standard deviation or frequency and percentage). Significant difference on univariate analysis (*p <* 0.05) is indicated by a *.

	West Coast (n = 148)	East Coast (n = 233)	Significance (*p* value)
	*Mean (standard deviation)*	*Mean (standard deviation)*	
**Age (years)**	8.9 (5.1)	12.5 (5.9)	.135
	*Frequency (percentage)*	*Frequency (percentage)*	
**Sex**	Mare 77(52.0)	Mare 96 (41.2)	.135
	Gelding 67(45.3)	Gelding 135(57.9)	.135
	Stallion 4(2.7)	Stallion 2(0.9)	.135
**Season**	Winter 33(23.3)	Winter 61(26.2)	.232
	Spring 41(27.7)	Spring 35(23.6)	.232
	Summer 50(35.8)	Summer 68(29.2)	.345
	Autumn 24(16.2)	Autumn 49(21.0)	.289
**Use**	Flat racing 31(20.9)	Flat racing 30(12.9)	.02*
	Barrel racing 60(40.5)	Barrel racing 18(7.7)	.005*
	Dressage 8(5.4)	Dressage 42(18)	.02*
	Hunter/jumper 24(16.2)	Hunter/jumper 58(24.9)	.121
	Other sport 4(2.7)	Other sport 11(4.7)	.135
	Pleasure 21(14.2)	Pleasure 74(31.8)	.081
**Complaint of cough**	70 (47.0)	171 (73.4)	.058*
**Complaint of exercise intolerance**	77 (52.0)	155(66.5)	.231
**Complaint of tachypnea or increased respiratory effort**	81 (54.7)	88 (37.8)	.058*

Bronchoaveolar lavage cytology differed between horses from the East and West on univariate analysis, with the most important finding being that horses from the West were significantly more likely to have birefringent particulates in the BAL lavage cytology than those from the East (Table 2). These particulates had varying shapes, from polygonal to round and rarely needle-like. The birefringent particulates were mostly less than 3 microns in greatest diameter, ranged from clear to pink to blue to dark brown or black, and were primarily contained within macrophages but also seen extracellularly. Whereas less than 10% of horses living in the East had visible intracytoplasmic birefringent particulates seen on polarizing microscopy, 41.5% of horses from the West had some evidence of intracytoplasmic birefringent particulates. Only 2.2% of horses from the East had more than rare evidence of birefringent particulates within alveolar macrophages, whereas 10.1% of horses from the West coast had 5–10% of macrophages containing birefringent particulates, and 10.8% of horses from the West had more than 10% of alveolar macrophages containing birefringent particulates (Table 2).

**Table 2:** Birefringence categorization and frequency/percentage of macrophages containing birefringent particulates.

Birefringence category	West Coast Horses	East Coast Horses	Chi-Square
	(n = 148)	(n = 233)	
0 (none seen)	88 (59.5%)	213 (91.4%)	P < 0.001
1 (extracellular and rare intracytoplasmic < 5% of macrophages)	29 (19.5%)	15 (6.4%)	P < 0.001
2 (occasional, 5–10% of macrophages), may also have occasional extracellular	15 (10.1%)	2 (0.9%)	P < 0.001
3 (many > 10% of macrophages), moderate-to-large numbers of extracellular	16 (10.8%)	3 (1.3%)	P < 0.001

Four horses, one from the East and the other three from the West, had large accretions of macrophages or bizarre giant multinucleated cells with high birefringent content (Fig 2B).

In addition, on univariate analysis, horses from the West had a significantly greater proportion of alveolar macrophages and eosinophils in the BAL cytology, whereas horses from the East had a significantly greater proportion of neutrophils. Horses from the East also had a significantly greater proportion of fungal elements, predominantly hay spores, found in the BAL cytology (61.8% v. 21.7%), whereas horses from the West were significantly more likely to have hemosiderophages (51.0% v. 28.8%) in the BAL cytology (Table 3).

**Table 3:** Bronchoalveolar lavage characteristics (mean and standard deviation or frequency and percentage). Significant difference on univariate analysis (*p <* 0.05) is indicated by a *.

	*West*	*East*	*Statistical Significance*
*BAL cytology proportions*	*Mean (standard deviation)*	*Mean (standard deviation)*	*p-value*
BAL Neutrophils (%)	10.9 (18.7)	27.1 (29.2)	.001*
BAL Mast Cells (%)	2.3 (2.1)	2.1 (3.9)	.291
BAL eosinophils (%)	1.5 (4.6)	0.24 (.93)	.001*
BAL alveolar macrophages (%)	54.9 (36.3)	44.8 (20.9)	.121
BAL lymphocytes (%)	33.7 (12.8)	26.9 (13.6)	.121
*BAL cytology–other findings*			
Giant cells	80 (54.1)	134 (57.5)	.659
Fungal elements	21 (14.3)	144 (61.8)	.001*
Epithelial cells	21 (42.0)	8 (19.5)	.021*
Hemosiderin	69 (46.6)	83 (35.6)	.029*

Further, on separate univariate analysis, there was no difference between horses with any degree of birefringence in the BAL and horses without with respect to clinical signs of cough, nasal discharge, poor performance, or increased respiratory rate (Chi-square), or for proportions of lymphocytes, alveolar macrophages, mast cells or eosinophils, but there was a significant difference between neutrophil proportions, with horses with evidence of birefringent particulates having significantly lower neutrophil proportions (13.99 +/-21.3 v. 22.1 +/-27.4, *p =* .006).

All historical and BAL data were subsequently included in a forward stepwise binary logistic regression model with presence of birefringent particulates found within alveolar macrophages as the outcome, based on standard criteria of entry and removal. Predictors that retained significance included geographical location of West (OR 8.0, CI [4.3–14.8], p< .001 and epithelial cells > 2% (OR 5.39, CI [1.99–14.63], p = .013). Using Nalgekerke R, 30.4% of the variability in the dependent variable–presence of birefringence in alveolar macrophages–was explained by our model.

## Discussion

We described and compared the clinical characteristics, cell proportions in BAL cytology, presence of birefringent particulates as well as fungal elements in the bronchoalveolar lavage cytology of horses living on the West v. the East coast of the United States, with several key findings. First, birefringent particulates were significantly more likely to be found in the BAL cytology of horses on the West coast, while fungal elements were more likely to be found in the BAL cytology of horses on the East Coast (Tables 2 and 3). Second, cell proportions differed between the two geographical locations, with greater proportions of neutrophils found in horses on the East Coast, and greater eosinophil proportions found in horses on the West coast (Table 2). Horses on the West Coast were also more likely to have hemosiderophages, but this finding was not retained in a multivariate model, likely because there were significantly greater proportions of barrel racing and flat racing horses in the horses from the West, and > 90% of racehorses experience exercise-induced pulmonary hemorrhage that is detected in BALF cytology as hemosiderophages; free red cells may also be seen in horses that have recently experienced alveolar hemorrhage [10] (Tables 1 and 3). In addition, univariate analysis showed that horses with any evidence of birefringent particulates had a lower neutrophil proportion in the BAL cytology but higher frequency of excessive epithelial cells. Using forward stepwise binary logistic regression with presence of birefringent particulates as the outcome measure, we found that the greatest risk factor was living on the West coast. The only other risk factor that was retained in our model was presence of higher-than-expected epithelial cells (more than 2-3/hpf). These findings suggest that geography is an important contributor to the likelihood of finding birefringent particulates in the BAL cytology. The documentation of birefringent particulates being the predominant foreign material in the BAL cytology of horses with suspicion of equine asthma on the West coast, whereas fungal elements are the predominant foreign material in the BAL cytology of horses with suspicion of equine asthma on the East coast, as well as differences in the cell proportions in the BAL cytology in these populations, is a novel finding.

While polarizing microscopy such as that used in this study can only confirm that a particulate is birefringent, the size and shape of the particulates we observed along with what is known about the makeup of inhaled geogenic dust can be used in conjunction with birefringence to make tentative identification of these substances. Silicates, which combine silica and other elements, are calculated to make up more than 90% of the earth’s crust [11]; accordingly, the disturbed land use dust to which agricultural workers are exposed, for example, has been documented to be almost 90% silicates [12]. The particulates that we observed in this study accord with other descriptions of silicates in biological specimens, being clear to blue-black color, size generally less than 5 microns, and variably shaped but often polygonal [13]; thus, a reasonable source for the birefringent particulates found in the respiratory secretions of horses is dust of crustal origin that contains silicates. Thomas et al. [14] noted that conversely to horses in the East, being stabled rather than living outdoors had a protective effect for horses with equine asthma living in the Western state, Texas, and similar to our findings, there was no influence of season. It is less likely, then that the cause of equine asthma in these outdoor-living horses was due to exposures to year-round geogenic dusts rather than to seasonal pollens and fungal loads as is seen in some southern states [1]. This argues that the role of geogenic dust–dust from the earth’s crust–may be important to the development of inflammatory airway diseases in horses living in arid regions and may further be an important indicator of exposure of humans to geogenic dust.

Geogenic dust poses a significant risk to cardiopulmonary health in arid regions of the world such as the west of the United States. Our finding of geographic habitation in the West the USA being the largest risk factor (OR 8.0) for presence of birefringence compatible with silicates seen in bronchoalveolar lavage fluid supports the contention that this geogenic dust is reaching the lower respiratory system of horses. As there has been a 240% increase in dust storms over the past 20-year period in the arid southwest of the USA due both to natural causes and human land disturbance [15], and computer modeling indicates that this geographical region will become increasingly drier in the coming decades, there is a very real risk that insult to the respiratory systems of both horses and humans due to geogenic dust will increase accordingly [16]. Although airborne geogenic dust has traditionally been observed to be highest in the summer [16], we found no effect of season in our study, and similarly recent analyses show that dust burdens are increasing in all seasons, further increasing the importance of better understanding of this environmental exposure [16].

Sustained workplace exposures to silica and silicate dusts are associated with the fibrotic pneumoconioses such as asbestosis, silicosis, and coal worker’s lung in humans [17]; reported multifocal-to coalescing fibrotic pulmonary disease along with a high macrophage burden of silica in hyraxes housed on silica play sand likely falls into this category [18]. Similarly, several reports of cases of naturally occurring severe, fibrotic pulmonary silicosis more similar to pneumoconioses in humans have been reported in horses in the Monterey-Carmel Peninsula in California presumably resulting from chronic inhalation of the high levels of endemic silica (cristobolite form) [19, 20]. Less severe, quotidian exposures to crustal airborne dusts, on the other hand, are implicated more frequently in cough, exacerbation of asthma or chronic obstructive pulmonary disease (COPD) in people [21], due to damage to alveolar walls and bronchiolar epithelium from both direct physical effects as well as oxidative stress with subsequent inflammatory reaction [22]. It is logical that individuals who spend large amounts of time outdoors in arid regions, including children [23] and agricultural workers, have been identified as having non-fibrotic respiratory diseases caused by or exacerbated by geogenic dust [24]. Non-fibrogenic silicosis, described as silica-laden macrophages (SLM), has recently been described in various animal species on the island of St. Kitts, with higher levels of silica found in species that are likely to graze or roll in dust [25]. Similarly, a study looking at 100 autopsies in 11 mammalian species and 8 avian species living in the southwest of the US showed mild pulmonary lesions despite silicate-laden macrophages; on energy-dispersive x-ray analysis, 95% of these were silicates [26]. Interestingly, studies in badgers, a species with a subterranean lifestyle, demonstrated plentiful silicate-laden alveolar macrophages, but a lack of pulmonary fibrotic change, which was attributed to the weathering of minerals encountered by the badgers or possibly an evolutionary adaptation to the environmental challenges [27]. No radiographic studies, biopsies, or autopsy results were available for any of the horses in this study, thus we have no evidence concerning existence of fibrogenic disease and cannot comment on the fibrogenic potential of birefringent particulates in this study; however, the presence of giant multinucleated cells containing a heavy load of birefringent material is consistent with chronic inflammation [28].

It was unsurprising that significantly larger numbers of horses on the East Coast had fungal elements, primarily single-cell spores compatible with conidiospores, in their BAL, as there is a large body of data to confirm the high levels of such spores in hay dust to which stabled horses are exposed [1]. This is the first study, however, to document the extent to which these spores reach the lower airways (over 61% of horses in the East Coast group), and it is comparable to that found in a European cohort of horses with a greater likelihood of being housed with straw bedding with high fungal spore content [29]. On the other hand, it was not expected that 21% of horses from the West Coast would also have evidence of fungal spores in the lower airways; this may be because of organic debris found in outdoor air [30] or may be due to hay feeding practices [1]. While no evidence was found of spores with the appearance of coccidiodes spherules, the presence of fungal species in the lower airways suggests that horses may be a useful sentinel species for exposure to Valley Fever, which is endemic to the Southwest, and appears to be increasing in prevalence in humans [15].

The finding that there are differences in BAL fluid cell proportions including neutrophils, eosinophils, and epithelial cells according to geographic location is also novel. While our study does not have data concerning length of time horses spent in the barn or what they were fed, it is reasonable to suppose that horses in the East of the USA are stabled at least part of the time, and indeed, our data (unpublished) show that approximately 90% of horses from the catchment area for our hospital and presenting for respiratory disease are stabled at least 8 hours a day. The high beta-glucans and endotoxins in the stable environment have been documented to cause airway neutrophilia in animals and people [1]. The increased proportions of eosinophils in BAL cytology of horses living on the West Coast is less easy to understand, but it may be due to enhanced immune responses in horses with pre-existing allergies, similar to that seen in a guinea pig model of allergic airway disease after exposure to inorganic particulates [31]. As the horses in this study were all presented for suspicion of equine asthma or, less commonly exercise-induced pulmonary hemorrhage (EIPH) in conjunction with equine asthma, it is unsurprising that horses from both coasts had evidence of airway inflammation.

Increased levels of epithelial cells have been found in the bronchoalveolar lavage cytology of people with acute silicosis, similar to our finding of high levels of epithelial cells (> 2-3/hpf) being a risk factor for presence of birefringence in the lung. Silicates and silica cause oxidative damage to the airways and alveolar spaces, resulting in visible damage to epithelial cells [32]. Agricultural dusts as mixed dust species have been shown to stimulate a higher concentration of oxidative species in alveolar macrophages, including hydroxide radicals and lipid peroxides, than even crystalline silica, thus explaining the ability of this geogenic dust to cause inflammation and fibrogenic disease in agricultural workers [24]. Inorganic particulates may independently elicit neutrophilic inflammation but may also act as carriers for endotoxin, beta-glucans, pollen, bacteria, fungi, and acidic molecules, and elicit neutrophilic inflammation in this way as well, but likely to a lesser extent than do the high levels of endotoxin and beta-glucans found in the stable environment [30].

There are multiple important limitations to this study. As this was a retrospective study assessing samples sent from clinicians around the country to a diagnostic cytology laboratory, it was not practical to assess cytokines in serum and BALF–this study was based on light microscopy alone. Similarly, samples are not processed and sent by veterinary practices in a manner that would allow accurate assessment of cellular expression of genes, which might have elucidated the pathogenesis of airway inflammation in these horses. Human asthma is recognized to have multiple endotypes, with atopic, or Th2-high allergic disease being only slightly more common than non-atopic [33] similarly, the plethora of endotypes in equine asthma is increasingly recognized, although non-atopic Th2-low is likely more common. Future studies including gene expression and serum and BALF cytokine analysis will be critical to a full understanding of the lower airway milieu in horses exposed to geogenic dust. A further limitation is that the author reading the slides (MRM) was not blinded to the location of the horses, as these were all clinical cases that required communication with the referring veterinarian. This was, however, a retrospective study, and no intention to analyze the data according to geography had been established when the cytologic analysis was performed, thus minimizing the potential bias. Unfortunately, there was no record of which horses were primarily stabled v. primarily kept outside; the assumption was that more horses in the West than in the East live outside due to weather conditions. As this study was not age-controlled, the influence of age alone on airway inflammation is potentially of interest, however, a study from the author’s group compared young and old horses and found no difference in arterial blood gases, measures of spirometry, respiratory resistance, or BAL cytology, making this a less likely confounder to our finding [34]. It is possible that the extracytoplasmic birefringent particulates may have been contaminants. Talc is commonly proposed as a possible contaminant, and as it is a silicate, has the potential to be confused for mineral dust exposure. However, talc has been eliminated from medical gloves for well over a decade, making this less likely. While it is still possible to purchase gloves powdered with cornstarch, the typical Maltese-cross appearance of cornstarch with polarizing microscopy makes this contaminant easy to identify. Many other biological materials are anisotropic, and thus exhibit birefringence, including urate crystals, cholesterol crystals, collagen, hair, some pollen grains, plant material, insect debris, cotton dust. The majority of these are either easily recognizable on microscopy or very unlikely to have contaminated a BAL sample. As the BALF may have been exposed to the air in stables where the procedure was usually performed, a more reasonable cause of contamination might be airborne dust containing mineral matter. We have attempted to reduce the risk of these particulates being confused for airborne exposure by documenting the presence of birefringent particulates within the cytoplasm of cells, thus further reducing the potential of contaminants.

In conclusion, our data show that birefringent particulates, compatible with mineral dust, especially silicates, are detectable using polarizing microscopy in BAL cytologies in horses presented for suspicion of respiratory disease. Further, these birefringent particulates are significantly more commonly found in horses residing in the semi-arid west of the USA and are associated with increased numbers of epithelial cells in the BAL cytology. These findings are in accordance with data from people living in arid and semi-arid conditions both in the west of the USA and in other parts of the world. While our data are insufficient to conclude that inhaled dust from the earth’s crust cause respiratory signs and airway inflammation or fibrogenic change in the horses in this study, the connection between similar findings in humans and other species and lower airway inflammation certainly supports the need for further research in this area. An increasing part of the world’s land mass is becoming arid or semi-arid, with an accompanying growing burden of airborne geogenic dust to which both humans and animals are exposed. The finding of birefringent particulates in lung cytology of horses primarily living in the West of the USA underscores the need to understand the effects of geogenic dust on the pulmonary system, as well as the need to decrease and avoid exposure to this dust.
